# Navigating Rough Waters: Global Shipping and Challenges for the North Range Ports

**DOI:** 10.1007/s10272-022-1047-4

**Published:** 2022-06-07

**Authors:** Jan Wedemeier, Lukas Wolf

**Affiliations:** Hamburg Institute of International Economics (HWWI), Fahrenheitstr. 1, 28359 Bremen, Germany

**Keywords:** F4, O11, R4

## Abstract

Ports and shipping have been in the spotlight in 2021 with surging demand, skyrocketing freight rates and week-long queues. This development stands against the background of the current global COVID-19 pandemic. Amid these disruptive waves, the North Range ports (Le Havre to Hamburg) face numerous challenges. This short analysis gives an overview of recent developments in international shipping and the potential for maritime transport as an early indicator for commodity trade. The article also explores the connection in import and export of the North Range ports to their respective countries and the EU. This article contributes to the extent to which maritime traffic data can be linked to economic data. Three long-term key challenges — sustainability, digitalisation and (de)globalisation — are discussed with a focus on the North Range ports as well as the newest effects of Russia’s war in Ukraine.

International shipping has been in rough waters over the past years. In 2019, the growth of global trade started to slow (Straubhaar, 2021), and after the COVID-19 pandemic emerged in late 2019 and hit most countries and their real economy worldwide for the first time in early to mid-2020 (Grömling, 2021). This caused significant disruptions in supply chains and logistical problems. As economies started to recover, demand rapidly surged in 2021 and international shipping has frequently been in the spotlight with tenfold increases in freight rates for containers, week-long queues in major ports as well as singular events such as the blockade of the Suez Canal (The Economist, 2021).

Considering these developments, we look at the North Range ports – the ports from Le Havre (France) via Rotterdam (the Netherlands) to Hamburg (Germany) – both their role as indicators for economic activity in the countries of the North Range and the European Union, as well as the major challenges that lie ahead.

* The authors would like to thank André Wolf (CEP), Claudia Wellenreuther (HWWI) and Sinja Rostalski (University of Göttingen) for helpful contributions, discussions and suggestions.

## The linkage between trade and ports

Precise indicators for economic development are always in demand but became much more important in light of the short-term disruptions of the pandemic. Delayed publication by statistical offices are critical in the majority of indicators for the economy, making real-time indicators difficult. For instance, the Federal Statistical Office of Germany has issued an early indicator for the economy based on freight rates of different transport modes: road, track, air and inland shipping. These transport indication data are connected to economic activity. There is, e.g. a high correlation with so-called experimental data between truck traffic on German motorways and the production of manufacturing. These experimental data, however, are not harmonised Europe-wide. Moreover, the degree of maturity, but also the quality of the results, differ from official statistics. To conclude, international shipping is strikingly lacking in these indicators. In this respect, a link between maritime transport and trade could close a gap to link these to economic data.

The immediate connection of shipping to nations' economies is in trade. In the EU, 80% of all imports and exports in volume and 50% in value are transported by ship (IHS, 2021). Especially countries that export and import internationally, such as Germany or the Netherlands, depend highly on global trade.

One established approach to measuring international trade volume is the Container Throughput Index (RWI/ISL, 2022). It includes data on container throughput from 91 international ports, which together account for around 60% of global container throughput. The index does not include goods shipped without containers, e.g. vehicles or bulk goods (Döhrn, 2019). It is published monthly with a delay of about two weeks. The index represents an estimate of shipping activity and is comparable to the so-called truck toll mileage index of the Federal Statistical Office (Destatis, 2022a). Looking at the development of the Container Throughput Index of the past years, a long-term increase to 117.9 can be seen until mid-2019 (base year 2015 = 100). Afterwards, the index slowly starts to decline, indicating a reduction of global trade before the pandemic. Once the pandemic hit the global economy, the index dropped to 106.8 in March 2020 and 105.6 in May 2020 – its lowest value since spring 2017. The two major slumps in March and May can be associated with the lockdown in China, and later in the rest of the world economy. Afterwards, the container index recovered and quickly surpassed pre-pandemic levels (ISL/RWI, 2022).

Apart from the slightly delayed Container Throughput Index, research has been put into using real-time shipping data as an immediate indicator for international shipping, and thus economic activity. Cerdeiro et al. (2020) from the IMF have set the methodological foundation, using the automatic identification system of ship vessels as a basis. Stamer (2021) has expanded on this, including not only nowcasting but accurate forecasting of economic trade with shipping data. Applying the model to Germany, the results show that shipping is more accurate in indicating trade volume than existing indicators and can be a valuable extension for early economic indicators for decision-makers (Stamer, 2021). Thus, the connection between shipping and economic activity of specific countries is being established and invites further research on the European and international levels.

### Throughput-flows of North Range ports

Seeing the potential of shipping in general as an early economic indicator for business cycles, brings into focus the role of the ports and their significance for their respective countries' economy. Looking at the North Range ports, we examine how far their container throughput may indicate their respective countries' economic trade volume. The major ports of the North Range from west to east are Le Havre, Zeebrugge, Antwerp, Rotterdam, Bremen/Bremerhaven and Hamburg. Occasionally, the Ports of Amsterdam, Wilhelmshaven and Dunkirk are also considered, but they are left out in the following analysis. The ports of Amsterdam and Dunkirk only have a small throughput relative to the main ports and tend to lose importance in goods handling, while Wilhelmshaven shows strong fluctuations and level effects due to the port extension. Listing the North Range ports by total trade volume in 1,000 tonnes in the second quarter of 2021, Rotterdam (151,478) has the highest volume, followed by the port of Antwerp (52,212), Hamburg (31,402), Le Havre (21,934), Bremen/Bremerhaven (15,902) and Zeebrugge (8,484) (Eurostat, 2022b).

Figures 1 and 2 provide an overview of the main North Range ports and their countries' and the EU's economic activity, using key statistical data provided by Eurostat (Eurostat, 2022a, 2022b; Destatis, 2022b). The analysis shows outflows, inflows, and total volume of the North Range ports compared to their countries' and the EU's export and import levels (Figure 3). The imports and exports have been transformed into an index based in the first quarter of 2018.

The smaller ports of the North Range, Zeebrugge and Le Havre, have highly fluctuating volumes with declines after the 2007-2008 global financial crisis and the COVID-19 pandemic. Apart from these business cycle crises, their port development is decreasing, running opposite to their countries' overall development in exports and imports. The downturn development indicates that these minor ports alone have no major significance for their country and are not able to indicate business economic activity on their own.

The German ports of Hamburg and Bremen/Bremerhaven had a similar development in Germany's foreign trade until 2015. But while the German imports and exports kept increasing, the ports disconnected from this development and fluctuated at a slightly decreasing level until the COVID-19 pandemic hit the European economy in 2020. This was also observed by Jessen-Thiesen (2022) when comparing port of Hamburg exports and imports to the EU. The sanctions against Russia due to the occupation of Crimea in 2014 (Kholodilin and Netunajev, 2016) may explain some of this decline in trade, but not the full stop in growth. As Germany's imports and exports kept increasing, the question arose about whether goods switched transport modes or were imported/exported through other (North Range) ports. Two of those may be the port of Wilhelmshaven or Gdansk, which have seen exploding volumes over the past years (Eurostat, 2022b).

The port of Antwerp mirrors nearly perfectly Belgium's trade volume, both in imports and exports. This does not change when comparing exports and imports from third countries to total volume. This underlines the major economic role of the port of Antwerp and the potential in us-

Figure 1
**The outflow of North Range ports and North Range countries, 2000-2021**
Index, 100=Q1 2018

Source: Eurostat (2022a, 2022b); Destatis (2022).

ing this connection as a business cycle indicator for the Belgian economy (see Figures 1 and 2).

The port of Rotterdam reflects the exports of the Netherlands to third countries. Both the Netherlands and the port's volumes are steadily increasing. In contrast, imports from third countries through Rotterdam decreased since the global financial crisis as the Netherlands' imports to third countries increased. In total imports and exports, the port of Rotterdam saw slight increases, though slower than the rest of the country (see Figures 1 and 2).

Shifting the focus to the European level, a strong connection can be seen between total exports of the North Range ports and the EU27. The development is similar in imports, although the EU27 grew faster, indicating that imports through other ports or means of transportation within the EU are becoming more important. Yet, both time series show a strong relation, indicating the importance of the North Range ports for the European economy (see Figure 3).

This short analysis has shown similar patterns for the major North Range ports and their countries' economies. Especially the port of Antwerp and Belgian foreign trade have shown strong similarities. At the same time, we have also highlighted a disconnection of the German ports to their country's trade and the unimportance of the minor ports of Zeebrugge and Le Havre on the national level. Unquestionably, the North Range ports are vital for the European Union and their countries' economies. It is also evident that the ports of Antwerp and Rotterdam had a stronger overall growth rate in recent years than the ports of Hamburg and Bremen/Bremerhaven, indicating the tough competition within the North Range ports and the competitive advantage of the former ports. It raises ques-

Figure 2
**The inflow of North Range ports and North Range countries, 2000-2021**
Index, 100=Q1 2018

Source: Eurostat (2022a, 2022b); Destatis (2022).

tions about the challenges that await the North Range ports in the upcoming years.

## Recent effects of the war in Ukraine on the North Range ports

Russia's ongoing war in Ukraine poses a new challenge and uncertainty for the North Range ports. Looking at the direct effects, only a small reduction in traffic is to be expected. The North Range ports have no significant connection to the Ukrainian economy, as there is only a negligible amount of shipping and hinterland transport to or from Ukraine. Rotterdam had the largest container trade volume with Ukraine of all the North Range ports with an average of one million tonnes per year over the past ten years. This represents 0.0025% of the total throughput of goods in Rotterdam. In all the other ports, this proportion is close to zero (Eurostat, 2022b). Thus, the effects of the war can be expected to be small. However, sanctions imposed upon Russia by the European Union and its member states, restricting specific goods, will have a much stronger impact on the North Range ports. All North Range ports handle a significant share of their volume with Russia. Especially in Hamburg and Rotterdam, the share of Russian volume of total goods throughput ranged from 10% to 15% between 2016 and 2020. The remaining ports have fluctuating trade with Russia from 1% to 17% of total handling (see Figure 4). The global economic upheavals due to the war are enormous, so that a further decline in trade is to be expected. A global supply crisis could grow from this and will imply geo-strategical changes (Jung, 2022; Kappel, 2022). In addition, inflation is gaining momentum due to recent shocks. The COVID-19 pandemic, which has been going on for two years, has disrupted the complex international supply chains in industrial production. The

Figure 3

**Inflow and outflow of North Range ports and EU27, 2000-2021**

Index, 100=Q1 2018

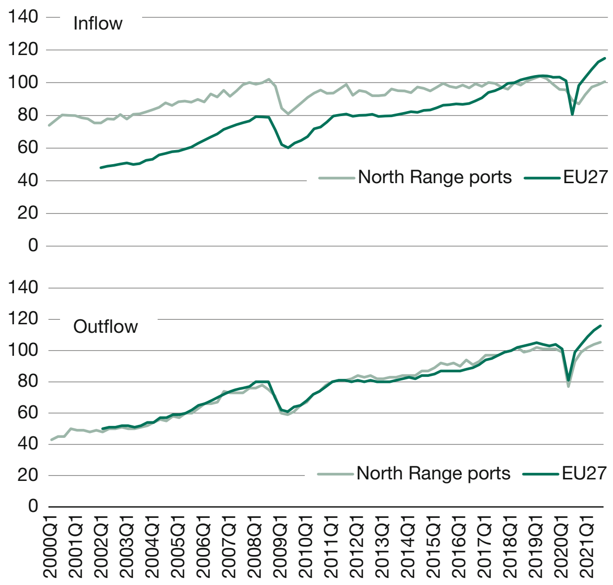

Source: Eurostat (2022a, 2022b); Destatis (2022).

recovery process hoped for in 2022 was interrupted by Russia's war in Ukraine. Not only were the supply chains running through Ukraine affected here, but the West's strict sanction policy against Russia is disrupting international trade (Berlemann et al., 2022). Currently, there is no end in sight, which implies a potential disruption to trade through the North Range ports.

## Further challenges for the North Range ports in the context of the COVID-19 shock

The North Range ports as major transport hubs are intertwined with Europe's economic activity. But they are faced with many immediate challenges, caused by the COVID-19 pandemic, as well as long-term transformations.

The effects of the pandemic-related recession on cargo handling in the ports cannot be foreseen. Maritime traffic has recovered quickly, however, after the initial phase of the COVID-19 pandemic. A further increase in freight volumes is expected worldwide in the coming years, which could have a positive effect on cargo handling volumes of the ports. It remains to be seen whether COVID-19 could accelerate pressing global trends in the maritime economy and thus require a massive restructuring of the industry. However, the key dimensions of sustainability, digitalisation, and (de)globalisation have played critical roles during the pandemic and will influence ports' development in the long-term perspective.

To further the discussion on the effects of the COVID-19 pandemic and the key issues for port development, Figure 5 presents the expected interactions in the short- and long-term qualitative forecast scenarios for the key dimensions with regards to port development. The correlations are estimated based on the preceding analysis and literature and do not represent an econometric relationship, nor do they present a comprehensive, all-encompassing forecast.

For sustainability, the short term has been dominated by a strong reduction in relevance. The immediate ef-

Figure 4

**Share of Russian good throughput in the North Range ports, 2016-2020**

Total volume in million tonnes

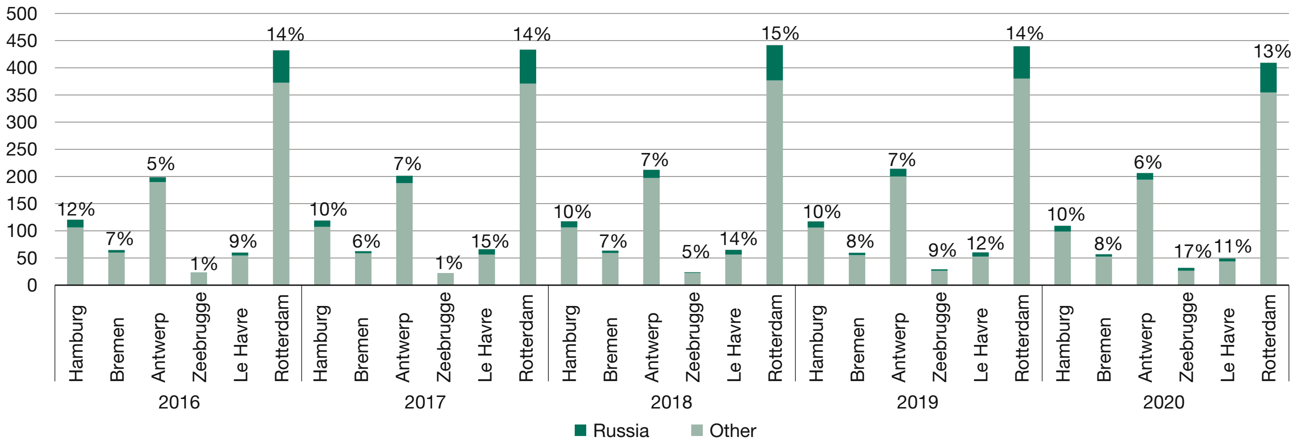

Source: Eurostat (2022b).

Figure 5
**Expected interactions of key dimensions with port development**

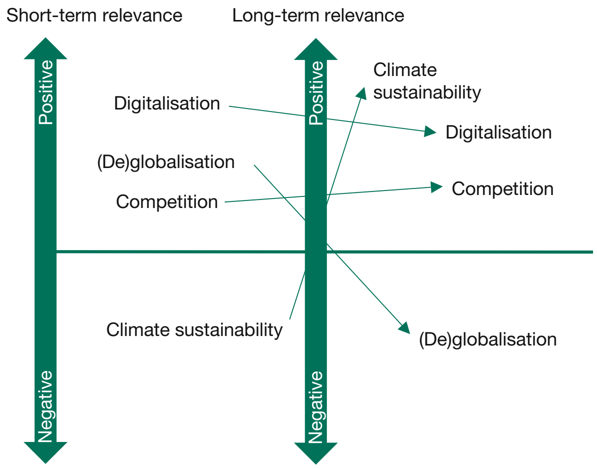

Source: Authors' own illustration.

fects of the COVID-19 pandemic might have decreased shipping and thus emissions, but the importance of the topic in society and the urgency for sustainable transformation processes were disrupted. Since then, sustainability has re-emerged as a key issue, especially for ports. Looking at the long-term perspective, ports have to achieve a green transformation of port infrastructure. This article differentiates the overarching themes of port sustainability into three dimensions. First, at the local level, ports need to adapt their infrastructure to operate (nearly) emission-free and to adapt to the changing environment. Additionally, waste management, pollution in air and water, and conflicts with natural habitats also play a role (Hossain et al., 2021). Secondly, in their role as maritime transport hubs, ports need to provide the necessary infrastructure for international shipping and hinterland transportation to run sustainably. Ports can create the stimulus for green shipping. However, the uncertain energy future for ships remains a problem (UNCTAD, 2021). Third, ports provide the necessary infrastructure for importing future in-demand resources, such as hydrogen or ammonia (EEA, 2021). The North Range ports have, for instance, begun a broad offense in implementing hydrogen strategies and infrastructure (for Hamburg, see HHLA, 2021; for Antwerp, Hydrogen Import Coalition, 2021; for Amsterdam, World Hydrogen Summit, 2022). European ports are particularly at the forefront of implementing sustainability measures and innovations (Hossain et al., 2021), supported by vast EU-funded projects (Nitt-Drießelmann and Wedemeier, 2021). Although sustainability adaptations come with costs and risks, they are increasingly pushed by national regulations and may present an advantage, turning the tide in the tough competition.

Digitalisation has benefited most from COVID-19 pandemic. Ports primarily faced disrupted supply chains and therefore needed to solve logistical problems (Mankowska et al., 2021; Notteboom and Haralambides, 2020). This underlined the importance and advantages of efficient digital processes, although implementing new digital infrastructure is difficult within a short time frame. In the long term, ports primarily transform their existing processes and implement new digital tools. Ports may use real-time data in all their infrastructure to enhance efficiency and decision-making. This gives ports the additional role of "information integrator and provider" (Heilig et al., 2017, 1347). New digital processes may also generate new data and information, which in turn can be used to analyse ports and improve port efficiency.

The COVID-19 pandemic had strong deglobalising effects with countries closing their borders, withholding critical resources and calling for a return to specific production facilities to Europe. These initially strongly affected ports rely on flourishing international trade and benefit from globalisation. But the resurgence of world trade in 2021 indicates that these developments will not have a lasting impact. In the long-term perspective, juxtaposed trends in recent years, e.g. the China-US trade war, Brexit, the COVID-19 pandemic and tensions over Ukraine, are on the rise, as are national foreclosures and deglobalising tendencies. There are growing numbers of geopolitical conflicts such as the war in Ukraine or Yemen, to name a few, and global changes such as the Belt and Road Initiative that could restructure competition between markets and ports. Global transport substitutes, power shifts and supply disruptions raise the geo-economic focus on the aspect of competition (Van de Putten et al., 2016). In contrast, (re-)established trade agreements such as the Regional Comprehensive Economic Partnership Agreement or the updated United States-Mexico-Canada Agreement enhance macro-regional and global trade (Flach et al., 2021). Despite calls for deglobalisation, a reversal of globalisation is not expected to produce the benefits of continuously great wage differentials between developing and industrial nations, specialisation, and the global interconnectedness through digitalisation (Dullien, 2018). However, a shift from physical to digital goods and services may take place (Straubhaar, 2021). If globalisation were to slow or be reversed to a certain extent, the immediate reaction for ports is a fall in demand and increased competition for cargo.

In summary, all three key issues ultimately affect the competition on throughput and hinterland shares, though port adaption to these dimensions differ. (De)globalisation is an exterior force setting the stage and specific adaption is difficult. The key issues of digitalisation and sustainability are general transformations in society and economy, which will force adaption, either voluntarily, by regulation or the expectations of customers. But these issues also allow North Range ports the opportunity to strengthen their positions and gain an edge in the already tight competition within the North Range and beyond.

## Conclusion

This analysis has demonstrated the importance of the North Range ports for the European economy and highlighted the advances in using maritime shipping as an early indicator. The discussed relations of ports to their respective countries' economy show similarities and imply the strong value of these relations. However, the graphical nature of the analysis can only give an overview and no conclusions on causal relations. A more sophisticated econometric analysis may provide insights into the importance of (North Range) ports and expand the knowledge on economic indicators. Additionally, the observed differences in the time series might be caused by unique events, such as massive shifts of shipping lines from one port to another or economic sanctions.

Looking ahead, ports will have to adapt to an array of challenges. In the short term, the lockdown in Shanghai and Russia's war in Ukraine will cause significant disruptions. Sustainability, digitalisation and globalisation are long-term issues, which will inevitably affect the development and competitiveness of the North Range ports. In the long run, the COVID-19 shock might weaken. It remains to be seen how the ports adapt individually and whether alliances will emerge to increase competitiveness and resilience. This may also allow sharing knowledge on key issues and jointly tackling transformation processes. However, Kappel (2022) states that the world situation has changed fundamentally as a result of the Russian war. Russia's aggressive move slows global growth on the brink of recovery from the COVID-19 pandemic. The growing supply crisis could herald a major global development. Western trading partners are looking for new leeway, but many states that are developing close to the EU and the United States are turning their backs and primarily looking for partnerships in fast-growing countries like China and India. The positioning of the North Range ports remains tense in this context.
